# Utility of pan-bacterial and pan-fungal PCR in endophthalmitis: case report and review of the literature

**DOI:** 10.1186/s12348-024-00419-9

**Published:** 2024-08-01

**Authors:** Carson W. Ercanbrack, Dania A. Rahal, Muhammad Z. Chauhan, Sayena Jabbehdari, Sami H. Uwaydat

**Affiliations:** 1 College of Medicine, University of Arkansas for Medical Sciences, Little Rock, AR USA; 2 Harvey and Bernice Jones Eye Institute, University of Arkansas for Medical Sciences, Little Rock, AR USA; 3 Department of Ophthalmology, Jones Eye Institute, University of Arkansas for Medical Sciences, 4301 W Markham Street # 523, Little Rock, AR 72205 USA

**Keywords:** Endophthalmitis, Intraocular infection, Chorioretinitis, pan-PCR, Next-generation sequencing, Quantitative PCR

## Abstract

**Background:**

Endophthalmitis is a clinical diagnosis but identification of the disease-causing agent or agents allows for a more tailored treatment. This is routinely done through intraocular fluid cultures and staining. However, culture-negative endophthalmitis is a relatively common occurrence, and a causative organism cannot be identified. Thus, further diagnostic testing, such as pan-bacterial and pan-fungal polymerase chain reactions (PCRs), may be required.

**Body:**

There are now newer, other testing modalities, specifically pan-bacterial and pan-fungal PCRs, that may allow ophthalmologists to isolate a causative agent when quantitative PCRs and cultures remain negative. We present a case report in which pan-fungal PCR was the only test, amongst quantitative PCRs, cultures, and biopsies, that was able to identify a pathogen in endophthalmitis. Pan-PCR has unique advantages over quantitative PCR in that it does not have a propensity for false-positive results due to contamination. Conversely, pan-PCR has drawbacks, including its inability to detect viruses and parasites and its increased turnaround time and cost. Based on two large retrospective studies, pan-PCR was determined not to be recommended in routine cases of systemic infection as it does not typically add value to the diagnostic workup and does not change the treatment course in most cases. However, in cases like the one presented, pan-bacterial and pan-fungal PCRs may be considered if empiric treatment fails or if the infective organism cannot be isolated. If pan-PCR remains negative or endophthalmitis continues to persist, an even newer form of testing, next-generation sequencing, may aid in the diagnostic workup of culture-negative endophthalmitis.

**Conclusion:**

Pan-bacterial and pan-fungal PCR testing is a relatively new diagnostic tool with unique advantages and drawbacks compared to traditional culturing and PCR methods. Similar to the tests’ use in non-ophthalmic systemic infections, pan-bacterial and pan-fungal PCRs are unlikely to become the initial diagnosis test and completely replace culture methods. However, they can provide useful diagnostic information if an infectious agent is unable to be identified with traditional methods or if empiric treatment of endophthalmitis continues to fail.

## Background

Endophthalmitis is an intraocular infection that results in considerable inflammation of intraocular structures and can result in permanent vision loss if not promptly treated. It is a clinical diagnosis, but intraocular fluid cultures are routinely Gram-stained and cultured to guide and tailor treatment. Since culture-negative endophthalmitis is common, pan-polymerase chain reaction (PCR) may offer an alternative diagnostic tool. Pan-PCR uses genome sequence as an input and returns the primers for a PCR assay capable of distinguishing the strains of interest.

Next-generation sequencing (NGS) is another emerging diagnostic tool that can be used. NGS, also called high-throughput or massively parallel sequencing, of microorganisms generally falls into one of two approaches. The first approach, targeted amplicon sequencing, employs target-specific primers for PCR amplification, which enables genomic regions of interest to be expanded and selectively sequenced using previously documented genomic references. The other strategy, whole-genome sequencing, depends on nontargeted library preparation and is typically employed when the microorganism is unknown. It can be applied to specimens for culture-independent pathogen identification and use in future NGS studies.

In this manuscript, we present a case where pan-PCR was able to identify a causative organism in the setting of endophthalmitis and review recent literature regarding pan-PCR in the diagnosis of intraocular infections, including endophthalmitis and infectious chorioretinitis.

## Main text

### Case report

**Fig. 1:**
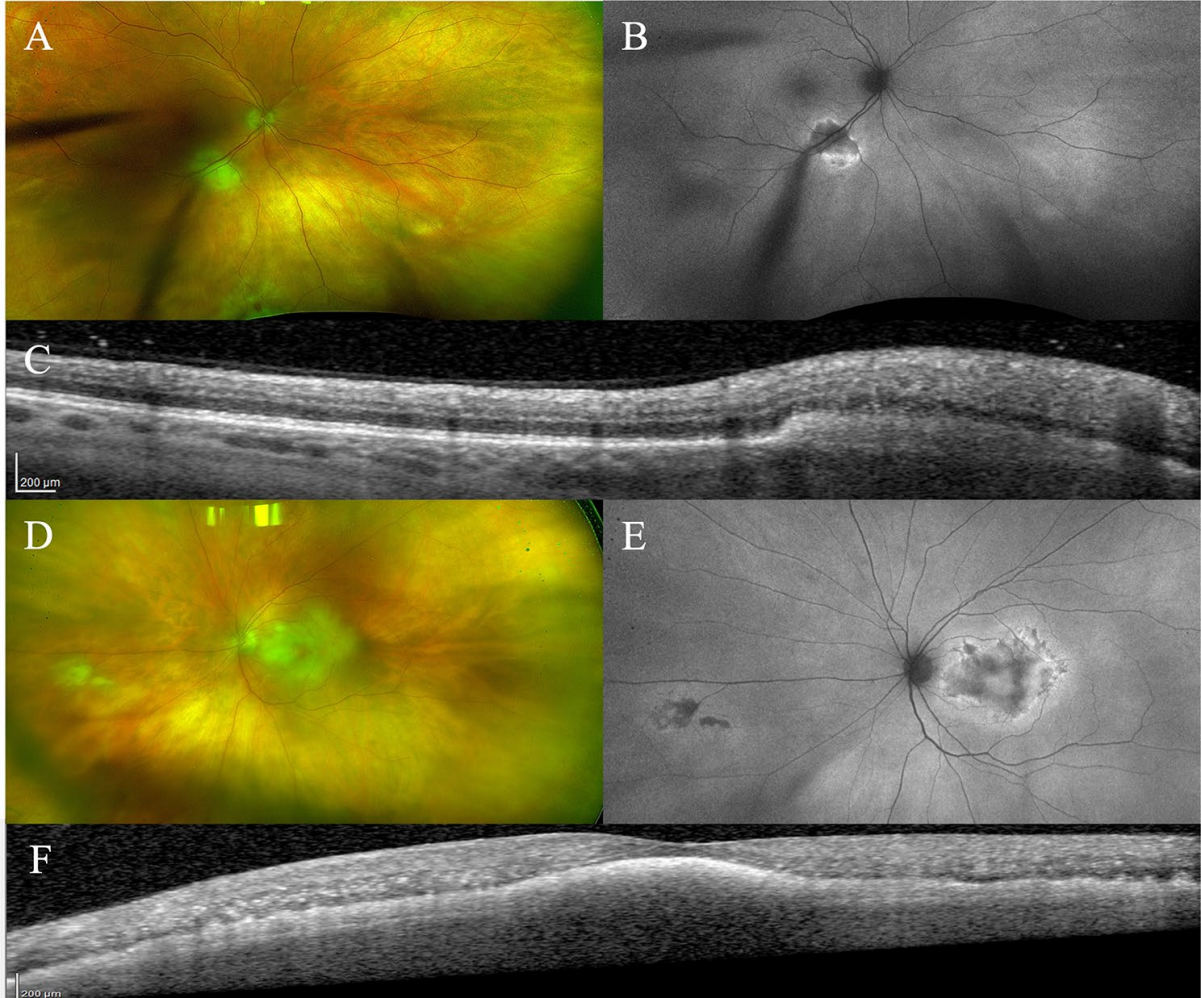
Widefield pseudocolor imaging (**A**) and fundus autofluorescence (**B**) of the right eye show subretinal infiltrates along the inferior arcade and inferior periphery. OCT B-scan through the lesion demonstrates sub-RPE infiltrates along the inferior arcade (**C**). Widefield pseudocolor imaging (**D**) and fundus autofluorescence (**E**) of the left eye show submacular infiltrates, with another patch in the nasal mid periphery. OCT B-scan through the macula arcade highlights sub-RPE infiltrates (**F**)

A 70-year-old male with a past medical history significant for relapsed acute myeloid leukemia presented to the clinic with blurry vision in his left eye and reported loss of central vision in the left eye. Visual acuity was 20/30 − 1 in the right eye and counting fingers in the left eye. Intraocular pressure was 12 mmHg in the right eye and 8 mmHg in the left eye. Anterior segment was significant for 1 + cells in the anterior chamber, and 2 + nuclear sclerosis in both eyes. On dilated examination of the right eye, a whitish subretinal lesion inferior to the macula with overlying hemorrhage was observed (Fig. 1A-B). Ocular coherence tomography (OCT) of the lesion showed sub-retinal pigment epithelium (RPE) infiltrates (Fig. 1C). Examination of the left eye revealed a similar whitish lesion over the macula with edema and hemorrhage noted (Fig. 1D-E). OCT of the left eye lesion displayed similar sub-RPE infiltrates (Fig. 1F). Quantitative PCR of the anterior chamber fluid of the left eye was negative for cytomegalovirus, herpes simplex virus 1 and 2, varicella-zoster virus, and *Toxoplasmosis gondii*. Magnetic resonance imaging (MRI) revealed chronic right centrum semiovale lacunar infarct and mild inflammation and enhancement along the posterior bilateral globes, concerning for an infectious or inflammatory etiology.

**Fig. 2:**
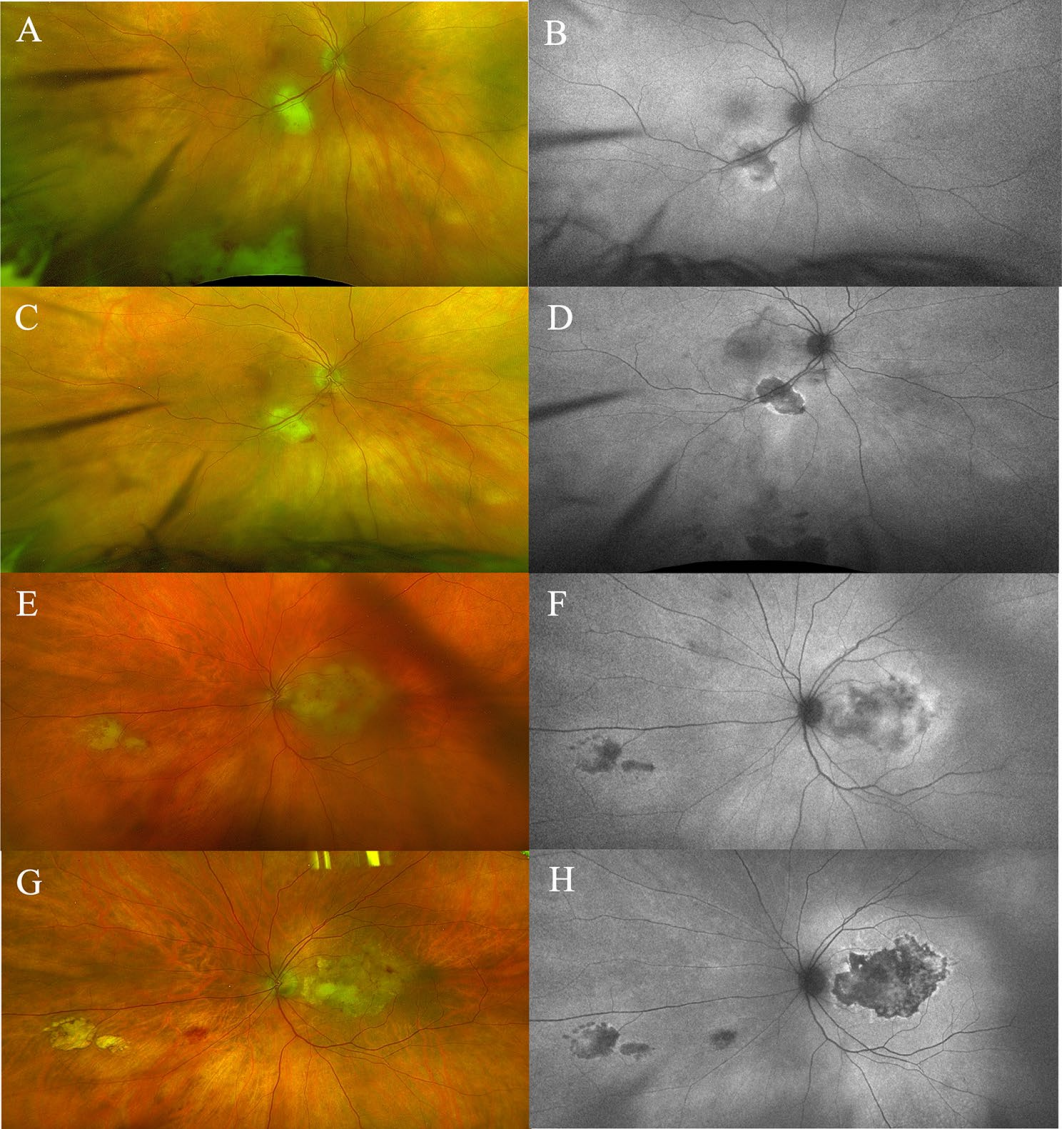
Widefield pseudocolor imaging (**A**) and fundus autofluorescence (**B**) of the right eye at the time of lesion enlargement and widefield pseudocolor imaging (**C**) and fundus autofluorescence (**D**) following treatment. Widefield pseudocolor imaging (**E**) and fundus autofluorescence (**F**) of the left eye at the time of lesion enlargement and widefield pseudocolor imaging (**G**) and fundus autofluorescence (**H**) following treatment. The lesions in both eyes show signs of regression with overlying RPE and retinal scarring after treatment

The patient had elevated beta-glucan levels (> 300 picograms per milliliter) and was started on vancomycin, ceftazidime, 300 milligrams, voriconazole twice daily, and amphotericin B (4 milligrams per kilogram) for potential fungal infection. In the following days, cytology revealed possible fungal elements in cerebrospinal fluid. A dilated fundus exam and Optos retinal imaging exam showed an enlargement of lesions in the right eye (Fig. 2A-B) and an enlargement of lesions with an additional area of hemorrhage in the left eye (Fig. 2E-F).

Nine days after the initial presentation, the patient underwent pars plana vitrectomy, vitreous biopsy, and a subretinal biopsy in the left eye. Biopsies were negative for neoplastic cells and did not have fungal elements with periodic acid shift plus diastase staining. Fungal and bacterial cultures, smears, and Gram stains of vitreous fluid were negative. Post-operatively, the lesion in the right eye (Fig. 2C-D) and the left eye (Fig. 2G-H) regressed in size with the formation of retinal scarring. Intravitreal injection of 0.1 milligrams/0.1 cc of voriconazole was administered, and lesion size was observed to decrease further in the left eye. Vision in the right eye began to improve at this time as well.

One week later, the patient received a stem cell transplantation. A dilated fundus exam at this time revealed that the macular chorioretinal lesion had increased in size. The patient received another 0.1 milligrams/0.1 cc of voriconazole intravitreal injection, and a vitreous fluid sample was sent to an outside reference laboratory for pan-fungal and pan-bacterial PCR. The post-transplant course was complicated by progressive renal failure and fluid overload, *Enterobacter* and *Enterococcus faecium* bacteremia, respiratory distress, and atrial fibrillation with rapid ventricular response. The patient’s white blood cell count dropped to 10 cells per microliter. The patient was discharged to hospice six weeks after the initial presentation. Following the patient’s death, results from the pan-fungal PCR revealed the detection of *Fusarium solani* in the vitreous sample.

### Review of recent literature

Gram-positive cocci are the most commonly implicated microorganism in endophthalmitis, being the attributed cause in about 90% of cases, with *Staphylococcus epidermidis* being the most common of the Gram-positive cocci. [1] While it is a clinical diagnosis, vitreous and aqueous samples are routinely cultured and Gram-stained to assist in categorizing the inciting organism and allowing for a more tailored approach to treatment. However, culture-negative endophthalmitis using aqueous and vitreous samples are relatively common, ranging from 40 to 70% in recent studies. [2] Another diagnostic challenge lies in the fact that aqueous samples from the anterior chamber are much easier to attain but have a much lower culture-positive rate at 22.5% than that of vitreous samples with a 54.9% culture-positive rate in the Endophthalmitis Vitrectomy Study conducted in 1997. [3] An additional pitfall of culture-based techniques in endophthalmitis is the fact that the microorganism that grows on the culture may not always be the disease-causing agent and a further, more comprehensive workup is still warranted even with a positive culture. [4]

Real-time PCR assays, also called quantitative PCRs, have been utilized as an adjunct to cultures to assist in the diagnosis of endophthalmitis. [1, 5, 6] PCR use presents unique challenges to physicians as the practitioner needs to have a clinical suspicion of a microorganism to determine the correct PCR test to administer. Furthermore, real-time PCR is very sensitive to contamination and may result in false positives. Additionally, it does not provide microorganism sensitivities like cultures do. [5] Yang et al. [7] have since purported that pan-PCR testing may have the ability to assist in the diagnosis of bacterial infections and provide an extra surveillance tool. Pan-PCR uses genome sequences as an input and returns the primers for a PCR assay capable of distinguishing the strains of interest. In such tests, computerized software utilizes existing genome sequences to identify a set of genes where the presence or absence of the genes provides the highest discrimination between species. Microorganisms’ unstable elements subject to rapid evolution (e.g., prophages and transposons) are filtered out by the algorithm, allowing for more reliability over time. The conserved sequences commonly tested in pan-bacterial PCR are the 16 S ribosomal subunit and the 5.8 S, 18 S, or 28 S ribosomal subunits in pan-fungal PCR. [8]

Pan-PCR techniques have been useful in recent studies with reported positive predictive values of 63.2% and 87.5% in diagnosing nontuberculous mycobacteria in suspected pulmonary or extra-pulmonary infections using pulmonary specimens and extra-pulmonary specimens, respectively, and 92.3% in diagnosing invasive fungal infections in patients with febrile neutropenia with blood samples. [9, 10] However, two large-scale prospective studies determined that routine use for diagnosis was not recommended, and the clinical utility of pan-PCR is generally low. Tkadlec et al. [11] reported in a retrospective study consisting of 1370 samples (75 heart valves, 151 joint tissue samples, 230 joint aspirates, 848 whole blood samples, and 66 cerebrospinal fluid samples) that about 70% of pan-PCR results were identical to culture results and that pan-PCR testing added value to the workup to only 173 cases. In another retrospective study of 585 samples by Khoury et al. [12], pan-PCR influenced management in only 27 cases.

While pan-PCR testing may not be recommended in other routine infectious etiologies, it may prove to be a valuable diagnostic test that can be used to aid in the treatment of endophthalmitis due to its relatively common culture-negativity. Unlike real-time PCR, pan-PCR testing does not have a propensity for false-positive results and is considered rare in such testing. [13] A prospective multicenter study consisting of 153 patients with clinically diagnosed acute and delayed-onset endophthalmitis evaluated the use and results of ocular fluid pan-bacterial PCR compared to culture. [1] In the study, 6 out of 22 culture-negative aqueous humor samples were positive with pan-bacterial PCR. Six out of 17 culture-negative vitreous samples had a positive pan-bacterial PCR. Additionally, the study showed that the positivity rates of pan-bacterial PCR and culture were not statistically different with both aqueous and vitreous samples (*P* = 0.6). After one dose of intravitreal 1 milligram of vancomycin and 2.25 milligrams of ceftazidime, 2 of 25 culture-negative aqueous samples were pan-bacterial PCR positive, and 16 of 27 culture-negative vitreous samples were pan-bacterial PCR positive. The increased positivity of pan-PCR compared to culture after intravitreal antibiotics may reflect the pan-PCR detecting DNA that persists after the organism has been killed. [8]

Pan-PCR’s clinical utility can be demonstrated in the case report presented within the manuscript. As stated in the patient’s clinical course, pan-fungal PCR was the only testing modality that was able to adequately identify a causative organism.

Although pan-PCR can be useful, it has a lower sensitivity and specificity than species-specific PCR and has a longer turnaround time, taking around 2–3 days for species identification compared to 2–3 h when using real-time PCR. [1] Additionally, the cost of pan-PCR averaged $133 and $616.08 for pan-fungal and pan-bacterial PCR, respectively, per test in 2020. [14–19] Furthermore, because of the increased turnaround time, bacteria associated with endophthalmitis with poorer prognoses (*Staphylococcus aureus* and *Streptococcus pneumoniae*) may be better identified using species-specific PCR before pan-PCR to enable earlier diagnosis and treatment initiation. [1] This conclusion is furthered by a study conducted by Cornut et al. [20] where *Staphylococcus*-specific PCR was positive in 7 negative pan-PCR cases. However, as previously stated, specific PCR is more prone to false positives, and thus, the 7 cases in which specific PCR was positive with a negative pan-PCR may reflect possible contamination as *Staphylococcus aureus* is a member of normal skin flora. The article does not mention if the PCR was positive for methicillin-sensitive or methicillin-resistant *Staphylococcus*.

Another drawback of pan-PCR testing lies in its inability to detect viruses and parasites. [21] Therefore, pan-PCR testing will unlikely be able to completely replace traditional culture and PCR methods, and further diagnostic testing may remain indicated if the physician is concerned about possible viral or parasitic etiologies of endophthalmitis despite negative pan-PCR.

**Fig. 3:**
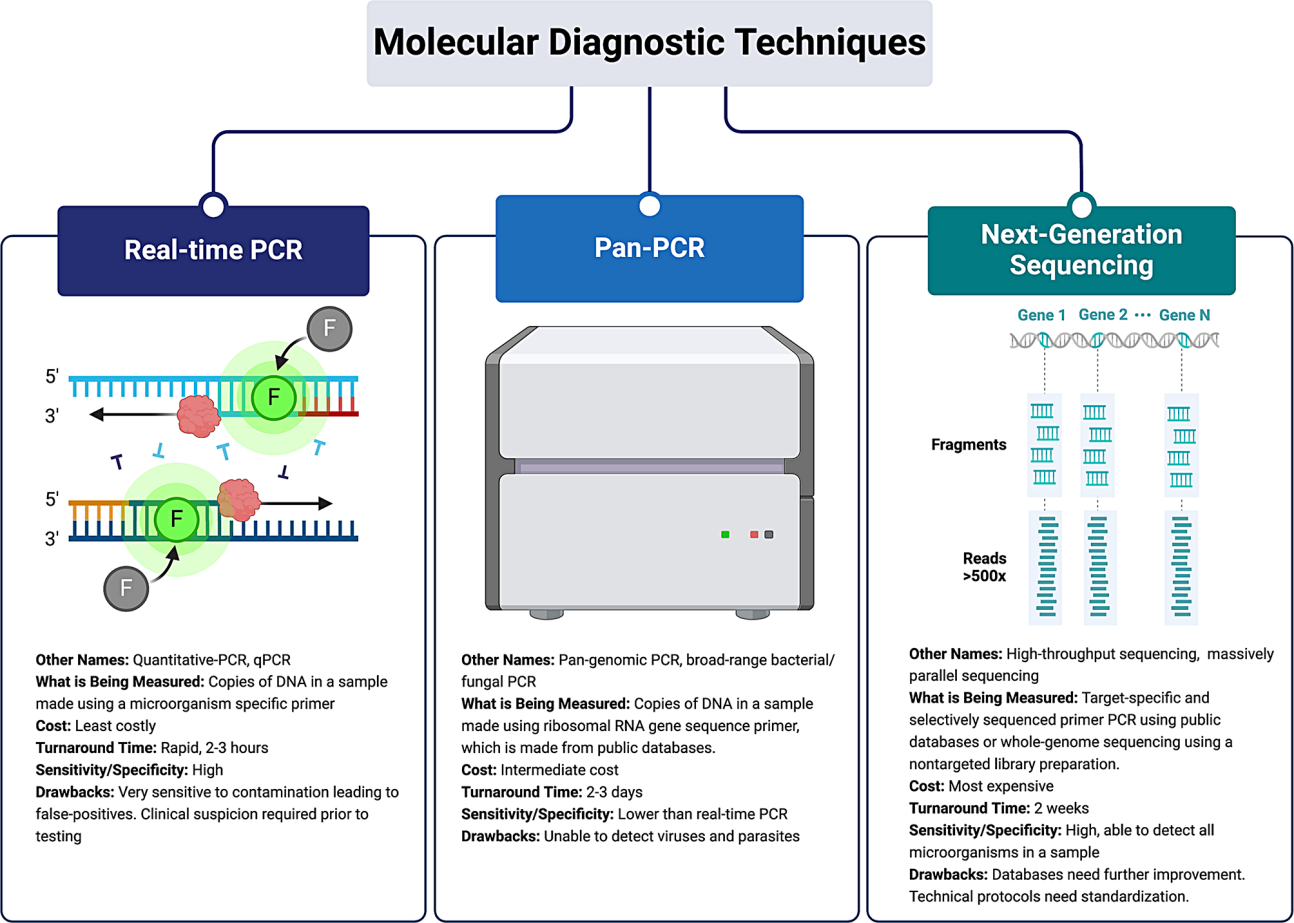
Illustration comparing real-time PCR, pan-PCR, and next-generation sequencing based on the item being measured, cost, turnaround time, sensitivity, and specificity. The graphic also denotes unique drawbacks in each of the methods

An additional emerging tool that may provide usefulness in the diagnosis and treatment of endophthalmitis when other traditional methods fail is next-generation sequencing (NGS), also termed high-throughput or massively parallel sequencing. NGS for microorganisms generally falls into one of two approaches. The first approach, targeted amplicon sequencing, employs target-specific primers for PCR amplification, which enables genomic regions of interest to be expanded and selectively sequenced using previously documented genomic reference databases. [22] The other strategy, whole-genome sequencing, depends on nontargeted library preparation and is typically employed when the microorganism is unknown. It can be applied to specimens for culture-independent pathogen identification and use in future NGS studies. [22] A retrospective study of 83 patients done by Cao et al. [23] reported a positive predictive value of 92.19% in diagnosing patients with NGS in cases of sepsis with an indeterminate site of infection or in patients for which it was difficult to get accurate pathogens. NGS is of particular interest as it has relatively high sensitivity and is capable of detecting all microorganisms within a sample, as exemplified in a reported case of chorioretinitis due to rhinovirus. [24, 25] An added benefit of NGS, compared to pan-PCR, is its ability to detect viruses and parasites in a given sample. [26] Going forward, NGS can be a useful tool for isolating pathogens that have not been previously associated with endophthalmitis or chorioretinitis and may be utilized when other diagnostic methods are non-revealing. However, NGS has its downfalls preventing routine use in microbiology, such as the improvement of reference databases, standardization of technical protocols, and reduction of cost ($1269-$2058 per test between years 2016–2019) and relatively slow turnaround time (about 2 weeks). [22, 27, 28] A graphic comparing quantitative PCR, pan-PCR, and NGS is displayed in Fig. 3.

## Conclusions

In conclusion, there are limitations to the use of pan-PCR as an initial testing modality in patients with endophthalmitis and chorioretinitis. Similar to systemic infections, pan-PCR may be unwarranted as an initial diagnostic test in endophthalmitis as the test may have low clinical utility and the test does not change treatment in a majority of cases when traditional methods are able to isolate a causative organism, as evidenced by two large prospective studies. Additionally, pan-PCR is unable to detect viruses and parasites that may be inciting endophthalmitis and, therefore, will not be able to replace traditional culture and PCR tests completely. Compared to real-time PCR, pan-PCR has a slightly longer turnaround time and is more expensive. Though the turnaround time is not significantly prolonged, the 2–3 days on average it takes to result may be more important when dealing with bacterial endophthalmitis associated with poorer outcomes and may be better utilized when such etiologies have been ruled out with traditional methods. Conversely, pan-PCR can provide useful insight into the inciting pathogen if initial cultures and PCR methods are negative or if empiric treatment of endophthalmitis continues to fail. Another advantage of pan-PCR is that it is less susceptible to contamination when compared to real-time PCR.

An additional testing modality that may be helpful in the future in determining the cause of endophthalmitis is next-generation sequencing. It can identify all intraocular pathogens within a sample and may have more clinical relevance, once its limitations, such as cost and turnaround time, are overcome.

## Data Availability

No datasets were generated or analysed during the current study.

## Abbreviations

PCR: Polymerase Chain Reaction
NGS: Next-Generation Sequencing
mmHg: millimeters of Mercury
OCT: Ocular Coherence Tomography
RPE: Retinal Pigment Epithelium

## References

[1] Kosacki J, Boisset S, Maurin M et al (2020) Specific PCR and quantitative real-time PCR in ocular samples from Acute and delayed-onset postoperative endophthalmitis. Am J Ophthalmol 212:34–42. 10.1016/j.ajo.2019.11.026 31770517 10.1016/j.ajo.2019.11.026

[2] Naik P, Gandhi J, Joseph J (2023) Recent advances and Ongoing challenges in the diagnosis of Culture negative endophthalmitis. Semin Ophthalmol 38(1):92–98. 10.1080/08820538.2022.2113101 35982639 10.1080/08820538.2022.2113101

[3] Barza M, Pavan PR, Doft BH et al (1997) Evaluation of Microbiological Diagnostic techniques in Postoperative endophthalmitis in the Endophthalmitis Vitrectomy Study. Arch Ophthalmol 115(9):1142–1150. 10.1001/archopht.1997.01100160312008 9298055 10.1001/archopht.1997.01100160312008

[4] Cheng P, Dong K, Kang Z et al (2022) Application of high-throughput sequencing technology in identifying the pathogens in Endophthalmitis. J Ophthalmol 2022:e4024260. 10.1155/2022/4024260 10.1155/2022/4024260 PMC9440830 36065285

[5] Joseph CR, Lalitha P, Sivaraman KR, Ramasamy K, Behera UC (2012) Real-time polymerase chain reaction in the diagnosis of Acute Postoperative Endophthalmitis. Am J Ophthalmol 153(6):1031–1037e2. 10.1016/j.ajo.2011.12.007 22381364 10.1016/j.ajo.2011.12.007

[6] Melo GB, Hofling-Lima AL, Bispo PJ, Pignatari AC (2011) Real-time pcr for the diagnosis of bacterial endophthalmitis. Invest Ophthalmol Vis Sci 52(14):5609 10.1167/iovs.10-5712 20702819

[7] Yang JY, Brooks S, Meyer JA et al (2013) Pan-PCR, a computational method for Designing bacterium-typing assays based on whole-genome sequence data. J Clin Microbiol 51(3):752–758. 10.1128/JCM.02671-12 23254127 10.1128/JCM.02671-12 PMC3592046

[8] Taravati P, Lam D, Van Gelder RN (2013) Role of Molecular Diagnostics in Ocular Microbiology. Curr Ophthalmol Rep 1(4):181–189. 10.1007/s40135-013-0025-1 10.1007/s40135-013-0025-1 PMC3885281 24416712

[9] Simon A, Onya O, Mazza-Stalder J, Nicod L, Gilbert G, Katia J (2019) Added diagnostic value of 16S rRNA gene pan-mycobacterial PCR for nontuberculous mycobacterial infections: a 10-year retrospective study. Eur J Clin Microbiol Infect Dis 38(10):1873–1881. 10.1007/s10096-019-03621-z 31313101 10.1007/s10096-019-03621-z PMC6778528

[10] Srinivas S, Kumari P, Gupta DK (2021) Utility of Panfungal PCR in the diagnosis of invasive fungal infections in febrile neutropenia. J Fam Med Prim Care 10(7):2533–2540. 10.4103/jfmpc.jfmpc_2325_20 10.4103/jfmpc.jfmpc_2325_20 PMC8415693 34568132

[11] Tkadlec J, Peckova M, Sramkova L et al (2019) The use of broad-range bacterial PCR in the diagnosis of infectious diseases: a prospective cohort study. Clin Microbiol Infect 25(6):747–752. 10.1016/j.cmi.2018.10.001 30321604 10.1016/j.cmi.2018.10.001

[12] Khoury N, Amit S, Geffen Y, Adler A (2019) Clinical utility of pan-microbial PCR assays in the routine diagnosis of infectious diseases. Diagn Microbiol Infect Dis 93(3):232–237. 10.1016/j.diagmicrobio.2018.09.016 30509499 10.1016/j.diagmicrobio.2018.09.016

[13] Chiquet C, Cornut PL, Benito Y et al (2008) Eubacterial PCR for bacterial detection and identification in 100 acute postcataract surgery endophthalmitis. Invest Ophthalmol Vis Sci 49(5):1971–1978. 10.1167/iovs.07-1377 18436828 10.1167/iovs.07-1377

[14] BRBPS - Overview: Broad Range Bacterial PCR and Sequencing, Varies. Accessed March 2 (2024) https://www.mayocliniclabs.com/test-catalog/overview/65058#Performance

[15] BROAD RANGE PCR (AFB, BACTERIAL, FUNGAL). Warde Medical Laboratory. Accessed March 2 (2024) https://wardelab.com/test/broad-range-pcr-afb-bacterial-fungal/

[16] Broad Range PCR | MLabs. Accessed March 2 (2024) https://mlabs.umich.edu/tests/broad-range-pcr

[17] Bacterial DNA (2024) Detection by PCR. Accessed March 2, https://testguide.labmed.uw.edu/view/BCTDNA?tabs=no

[18] Garnham K, Halliday CL, Kok J et al (2020) Knowledge at what cost? An audit of the utility of panfungal PCR performed on bronchoalveolar lavage fluid specimens at a tertiary mycology laboratory. Pathol (Phila) 52(5):584–588. 10.1016/j.pathol.2020.03.013 10.1016/j.pathol.2020.03.013 32576387

[19] Aggarwal D, Kanitkar T, Narouz M, Azadian BS, Moore LSP, Mughal N (2020) Clinical utility and cost-effectiveness of bacterial 16S rRNA and targeted PCR based diagnostic testing in a UK microbiology laboratory network. Sci Rep 10(1):7965. 10.1038/s41598-020-64739-1 32409679 10.1038/s41598-020-64739-1 PMC7224368

[20] Cornut PL, BOISSET S, ETIENNE J et al (2012) Specific pcr detection of Staphylococcus and Streptococcus in Endophthalmitis. Invest Ophthalmol Vis Sci 53(14):1688

[21] Naureckas Li C, Nakamura MM Utility of Broad-Range PCR sequencing for infectious diseases clinical decision making: a Pediatric Center Experience. J Clin Microbiol 60(5):e02437–e02421. 10.1128/jcm.02437-21 10.1128/jcm.02437-21 PMC9116169 35400176

[22] Lefterova MI, Suarez CJ, Banaei N, Pinsky BA (2015) Next-generation sequencing for infectious Disease diagnosis and management: a report of the Association for Molecular Pathology. J Mol Diagn 17(6):623–634. 10.1016/j.jmoldx.2015.07.004 26433313 10.1016/j.jmoldx.2015.07.004

[23] Cao X, guang, Zhou S, sheng, Wang C, Jin yan, Meng K (2022) H dong. The diagnostic value of next-generation sequencing technology in sepsis. *Front Cell Infect Microbiol*. ;12. Accessed February 4, 2024. https://www.frontiersin.org/articles/10.3389/fcimb.2022.899508 10.3389/fcimb.2022.899508 PMC9518011 36189371

[24] Deshmukh D, Joseph J, Chakrabarti M et al (2019) New insights into culture negative endophthalmitis by unbiased next generation sequencing. Sci Rep 9(1):844. 10.1038/s41598-018-37502-w 30696908 10.1038/s41598-018-37502-w PMC6351655

[25] Bourdin A, Tout ée A, Fardeau C (2023) Intravenous immunoglobulins for bilateral Retinochoroiditis in Rhinovirus infection: a Case Report. Ophthalmic Surg Lasers Imaging Retina 54(12):720–722. 10.3928/23258160-20231019-02 38113358 10.3928/23258160-20231019-02

[26] Chen H, Fan C, Gao H et al (2020) Leishmaniasis diagnosis via Metagenomic Next-Generation sequencing. Front Cell Infect Microbiol 10. 10.3389/fcimb.2020.528884 10.3389/fcimb.2020.528884 PMC7538539 33072623

[27] Lee Y, Clark EW, Milan MSD et al (2020) Turnaround Time of plasma next-generation sequencing in thoracic oncology patients: a quality improvement analysis. JCO Precis Oncol 41098–1108. 10.1200/PO.20.00121 10.1200/PO.20.00121 PMC7529535 33015530

[28] Desai K, Hooker G, Gilbert K, Cropper C, Metcalf R, Kachroo S (2021) Real-world trends in costs of next generation sequencing (NGS) testing in U.S. setting. J Clin Oncol 39(15suppl):e18824–e18824. 10.1200/JCO.2021.39.15_suppl.e18824 10.1200/JCO.2021.39.15_suppl.e18824

